# Individualized cognitive behavioral therapy for chronic pain in Japan: study protocol for a multicenter randomized controlled trial

**DOI:** 10.1186/s13063-025-09153-6

**Published:** 2025-11-11

**Authors:** Hiroki Hosogoshi, Kazunori Iwasa, Takaki Fukumori, Yuriko Takagishi, Yoshitake Takebayashi, Yukino Tairako, Yuki Oe, Tomonori Adachi, Kiyoka Enomoto, Satoshi Yokoyama, Jiro Kurata, Ayano Matsui, Hiroyuki Nishie, Hitoaki Sato, Noriyo Takahashi, Keisuke Watanabe, Aki Fujiwara, Atsuo Yoshino, Ayaka Toyota, Masaru Horikoshi, Masahiko Shibata

**Affiliations:** 1 Faculty of Sociology, Kansai University, Suita, Japan; 2 National Center for Cognitive Behavior Therapy and Research, National Center of Neurology and Psychiatry, Kodaira, Japan; 3 Graduate School of Sustainable System Sciences, Osaka Metropolitan University, Sakai, Japan; 4 Graduate School of Technology, Industrial and Social Sciences, Tokushima University, Tokushima, Japan; 5 Department of Health Risk Communication, Fukushima Medical University School of Medicine, Fukushima, Japan; 6 Faculty of Health Sciences, Kyorin University, Mitaka, Japan; 7 Department of Neuropsychiatry, Kyorin University School of Medicine, Mitaka, Japan; 8 Graduate School of Human Development and Environment, Kobe University, Kobe, Japan; 9 Pain Management Clinic, Shiga University of Medical Science Hospital, Otsu, Japan; 10 United Graduate School of Child Development, University of Osaka, Suita, Japan; 11 Faculty of Humanities, Niigata University, Niigata, Japan; 12 Department of Anesthesiology and Perioperative Medicine, Jikei University Graduate School of Medicine, Nishi-Shimbashi, Japan; 13 Department of Orthopaedic Surgery, National Center Hospital of Neurology and Psychiatry, Kodaira, Japan; 14 Department of Anesthesiology and Intensive Care Medicine, Kawasaki Medical School Hospital, Kurashiki, Japan; 15 Division of Anesthesiology, Department of Surgery Related, Kobe University Graduate School of Medicine, Kobe, Japan; 16 Multidisciplinary Pain Center, Senriyama Hospital, Toyonaka, Japan; 17 Department of Anesthesiology, Nara Medical University, Kashihara, Japan; 18 Center for Brain, Mind and KANSEI Sciences Research, Hiroshima University, Hiroshima, Japan; 19 Department of Health Science, Naragakuen University, Nara, Japan

**Keywords:** Chronic pain, Cognitive behavioral therapy, Psychotherapy, Multicenter randomized controlled trial, Study protocol, Quality of life, Psychosocial outcomes, Mediators and moderators, Japan

## Abstract

**Background:**

Chronic pain imposes a substantial burden on individuals and society. Cognitive behavioral therapy for chronic pain (CBT-CP) has proven effective internationally, but randomized evidence in Japan is scarce. The first Japanese trial was a single-center, 16-session videoconference program that prioritized pain intensity. In contrast, this multicenter trial tests a brief, face-to-face, individualized CBT-CP program with quality of life (QoL) as the primary outcome.

**Methods:**

This open-label, randomized, parallel-group superiority trial enrolls 60 adults aged 20–80 years with chronic pain persisting ≥ 3 months and EQ-5D-5L scores ≤ 0.80, indicating reduced QoL. Participants are randomized 1:1 to treatment as usual (TAU) plus an eight-session CBT-CP program or to a waitlist control receiving TAU only. The manualized intervention includes psycho-education, relaxation, activity pacing, and cognitive restructuring, delivered weekly face-to-face, with the goal of completing eight sessions within 14 weeks. If necessary, sessions may extend beyond the 15th week; however, the post-assessment is always conducted at 15 ± 2 weeks irrespective of intervention timing. Therapists meet prespecified eligibility criteria and receive structured training, supervision, and fidelity monitoring. The primary outcome is QoL (EQ-5D-5L) at 15 ± 2 weeks. Secondary outcomes include pain intensity, disability, depressive symptoms, catastrophizing, fear of movement, pain self-efficacy, and health-related QoL assessed by the SF-12. These outcomes, together with prognostic risk and somatic symptom burden, will also be examined as potential mediators or moderators. The intervention group undergoes a 27 ± 2-week follow-up to explore mid-term durability in a single-arm pre/post analysis. Analyses follow the intent-to-treat principle using linear mixed models, with effect sizes and confidence intervals reported; multiplicity will be considered in interpreting secondary outcomes. Blinding of participants and therapists is not feasible.

**Discussion:**

This trial will clarify the added value of a brief, fidelity-assured, face-to-face CBT-CP program in Japan, where implementation remains limited. Findings are expected to guide clinical adoption, workforce training, and dissemination, while also generating hypotheses about for whom and through which processes CBT-CP may confer benefit.

**Trial registration:**

University Hospital Medical Information Network Clinical Trials Registry, UMIN000042798. Registered on 21 December 2020, https://center6.umin.ac.jp/cgi-open-bin/ctr_e/ctr_view.cgi?recptno=R000048858.

## Introduction

### Background and rationale {6a}

Chronic pain typically lasts more than 3 months [1, 2] and is defined as “an unpleasant sensory and emotional experience associated with, or resembling that associated with, actual or potential tissue damage” [3]. Chronic pain causes various problems, including behavioral problems owing to decreased activities of daily living (ADL) [4], emotional problems such as depression and/or anxiety [5], cognitive problems such as catastrophizing [6], and general problems such as decreased quality of life (QoL) [7, 8]. The prevalence of chronic pain is 11–40% in the USA [9], 35–51% in the UK [10], and 13–26% in Japan [11]. The annual loss of social productivity due to chronic pain is estimated to be $560–635 billion in the US [12] and $19.9 billion in Japan [8]. Thus, it has an enormous impact on individuals and society, and managing it is an important issue.

The ultimate goal of chronic pain treatment is not to attain pain-free conditions but to manage the pain to improve QoL and/or ADL [13, 14]. Cognitive behavioral therapy (CBT) is a psychological approach to managing the psychosocial factors that interact with chronic pain, and many clinical trials have demonstrated its efficacy and cost-effectiveness for chronic pain. A Cochrane Collaboration systematic review found that CBT for chronic pain (CBT-CP) significantly reduced pain intensity, disability, and distress compared to standard treatment (standardized mean difference (SMD) = − 0.22, − 0.32, − 0.34) in adult patients and maintained the effects for several months (SMD = − 0.16, − 0.21, − 0.25) [15]. In a randomized controlled trial (RCT) of CBT-CP in patients with subacute and chronic low back pain, those who received CBT-CP experienced reduced pain and improved ADL and QoL after 12 months, and their treatment costs were half to one-fifth of those undergoing acupuncture, exercise therapy, or manual therapy [16]. Consequently, CBT-CP has often been described as the gold standard psychological approach for individuals with different pain problems [17].

In Japan, although CBT-CP has been recommended in various Japanese guidelines based on evidence from Western studies, its validation has been delayed and its prevalence remains limited [14]. Only a small number of clinical trials on CBT-CP have included Japanese participants, mainly consisting of retrospective studies comparing responders and non-responders to treatment with insufficient assurance of therapist quality [18, 19]; a practice report of exercise therapy combined with psycho-education of CBT with a self-administered text [20]; and a few single-arm, pre/post studies without control groups [21–23]. Reflecting this lack of research, in medical practice in Japan, about 70% of treatments are massage, medication, and physical therapy; about 25% are acupuncture, prosthesis therapy, block therapy, and surgery; and about 4–7% fall into other categories, only a portion of which are psychological approaches, typically delivered by psychologists, including CBT-CP [24, 25]. This overall insufficiency in both research and practice is underscored by the latest Japanese guideline (2021), which explicitly states that although the implementation of CBT-CP can be recommended, a suitable environment, including the availability of trained psychologists, is not yet in place and the implementation system in Japan requires prompt development [14]. Subsequently, the first RCT of CBT-CP in Japan was reported in 2021 [26]. However, it was performed as a single-center study with a small sample size of 30 patients, and the primary outcome was pain itself, rather than QoL, considered the highest priority [13, 14]. In addition, although the RCT employed a 16-session program that included many modalities to be applicable to a wide range of patients, it is also important to develop a short-term program with selected modalities and to examine its efficacy, as a shorter program would be more acceptable in medical practice in Japan. To enhance the generalizability of CBT-CP in Japan, developing short-term CBT-CP programs and, in particular, rigorously testing them in large-scale multicenter trials with QoL as the primary outcome will be essential steps. Alongside this primary focus on QoL, secondary outcomes will include a range of psychosocial variables (e.g., pain severity, disability, and mental health), allowing assessment of broader effects of CBT-CP on patients’ functioning and well-being. In addition, assessing mid-term outcomes at follow-up will provide insights into whether treatment effects are sustained beyond the immediate post-intervention period.

Previous reviews have highlighted that the mechanisms of change in CBT remain insufficiently understood and that carefully designed studies are needed to clarify the role of potential mediators and moderators [27]. In this context, exploratory analyses of such variables may provide useful insights, particularly when QoL is the primary outcome. In this trial, however, these analyses remain exploratory, while the primary objective is to establish whether CBT-CP can improve QoL in Japanese patients.

Additionally, to rigorously evaluate the efficacy of CBT-CP, it is essential that interventions are appropriately conducted. Some previous clinical trials noted insufficient assurance in this regard [15]. Therefore, it is necessary to clearly indicate how therapists were trained in advance and to monitor whether the content of CBT-CP delivered is appropriate and whether the therapeutic process is adequate. Ensuring these procedures and treatment fidelity is a prerequisite for verifying the efficacy of CBT-CP, while also providing a reliable foundation for subsequent exploratory analyses of its mechanisms.

No formal patient or public involvement was implemented in the design, conduct, or reporting of this trial. However, the trial was designed with a primary focus on patient-reported outcomes, and experiences from the preceding pilot study with Japanese patients informed the refinement of the program and trial procedures.

### Objectives {7}

The primary objective of this multicenter RCT is to examine whether an eight-session individualized CBT-CP program, in addition to treatment as usual (TAU), improves QoL in Japanese patients with chronic pain compared with a waitlist control group receiving TAU only. The primary outcome is QoL, assessed using the EuroQol five-dimensional questionnaire five-level (EQ-5D-5L), measured at baseline and at 15 ± 2 weeks.

Secondary objectives are to evaluate the effects of CBT-CP on related psychosocial factors, and to assess mid-term effects in a single-arm pre/post comparison at 27 ± 2 weeks.

Exploratory objectives are to examine whether psychosocial variables, participant characteristics, and process measures function as potential mediators or moderators of treatment response.

### Trial design {8}

The study uses a multicenter, open-label, randomized, parallel-group superiority design and will involve 60 patients aged 20–80 years with chronic pain. Initial evaluation is conducted after obtaining informed consent, and eligible patients undergo a baseline assessment within 4 weeks and are enrolled as participants. Participants are randomized in a 1:1 allocation ratio to the intervention group (TAU plus an eight-session CBT-CP program) or the waitlist control group (TAU only). Participants in the waitlist group continue their usual care without CBT-CP until the post-assessment at 15 ± 2 weeks after randomization, after which they are offered the intervention.

## Methods: participants, interventions, and outcomes

### Study setting {9}

This multicenter study is being conducted at Kansai University, with which the principal investigator (HH) is affiliated, and at seven hospitals that will recruit participants and implement the intervention to reach the target sample size: the National Center of Neurology and Psychiatry Hospital in Tokyo, Jikei University Hospital in Tokyo, Hiroshima University Hospital in Hiroshima, Senriyama Hospital in Osaka, Kawasaki Medical School Hospital in Okayama, Kobe University Hospital in Hyogo, and Nara Medical University Hospital in Nara, all located in Japan.

### Eligibility criteria {10}

#### Inclusion criteria (all must be met)

- Chronic pain persisting for at least 3 months
- EQ-5D-5L ≤ 0.80
- Disability attributable to chronic pain
- Age 20–80 years
- Able to understand the study and provide written informed consent

#### Exclusion criteria (any of the following)

- Currently receiving other psychotherapy or participating in another clinical trial
- Previous receipt of CBT for chronic pain
- Scheduled to receive medical examinations or treatments that may interfere with participation or outcomes, specifically: (1) interventional pain procedures such as surgeries, orthopedic procedures, nerve blocks, or trigger point injections; (2) prescription of medical narcotics, even if type and dosage are fixed; and (3) intravenous infusion for pain relief.
- Pain with an organic cause requiring immediate medical attention
- Chronic pain mainly consisting of headache (primary or secondary). Headache has traditionally been reviewed separately and is generally managed through distinct treatment pathways and outcome domains [15, 27], making its inclusion less consistent with the aims of this trial.
- Chronic pain due to surgery or external injury
- Currently receiving compensation or benefits related to litigation for chronic pain
- Alcohol or substance use disorder
- Manic episodes or any psychotic disorder
- Severe suicidal ideation
- Cognitive impairment that precludes participation in CBT
- Insufficient ability to communicate, read, or write in Japanese
- Pregnancy
- Any other condition deemed unsuitable by the attending physician

#### Therapists

To ensure the quality of the intervention, the requirements for therapists in this study are as follows: (1) medical professionals such as physicians, clinical psychologists, nurses, physical therapists, and occupational therapists with at least 2 years of experience in treating chronic pain or CBT; and (2) completion of at least one patient’s therapy that was fully supervised by one of the supervisors (MH, HH, KI, TF, Y. Takagishi) and subsequent approval as a CBT-CP practitioner. At the beginning of recruitment in December 2020, eight psychologists (HH, KI, TF, Y. Takagishi, YO, Y. Tairako, TA, KE; three women and five men; three with an M.A. and five with a PhD; 3–15 years of clinical experience; 5–15 CBT-CP cases after certification) served as therapists, all of whom held psychotherapy certifications (clinical psychologist, a private qualification recognized by the Foundation of the Japanese Certification Board for Clinical Psychologists, and certified public psychologist, a national certification in Japan). In February 2023, a licensed psychologist (SY; a man, PhD, 8 years of clinical practice experience, and two fully supervised CBT-CP cases after certification) was additionally enrolled as a therapist.

### Who will take informed consent? {26a}

At each participating hospital, the collaborating physician (JK, AM, HN, HS, NT, KW, AF, AY, MS) is primarily responsible for identifying eligible patients. These physicians recruit participants either from their own patient population, through referrals from colleagues or affiliated institutions, or when patients themselves contact the hospital after finding information about the study on the study website. When a patient expresses interest, the physician and/or a designated research collaborator provide both verbally and in writing explanations of the study objectives and procedures using the approved patient information sheet. If a therapist provides the explanation alone, this is always done under the supervision of the physician. Informed consent is usually obtained directly by the recruiting physician; however, in some cases, it is obtained by a trained therapist (HH, KI, TF, Y. Takagishi, Y. Tairako, YO, TA, KE, SY) or by a research assistant (AT) under the physician’s supervision. After written consent is obtained, the patient undergoes an initial evaluation during which eligibility is confirmed. If the participant has an attending physician at another hospital, a written summary of the study procedures is also sent to the attending physician to request cooperation.

### Additional consent provisions for collection and use of participant data and biological specimens {26b}

No biological specimens are collected in this trial, and no incidental findings are expected. Regarding participant data, the consent procedure includes provisions for possible secondary use. Specifically, anonymized data obtained in this study may be used for ancillary analyses by the investigators or shared in public databases or with other researchers for purposes such as replication or meta-analysis. Any such use will require prior approval by the relevant institutional ethics committee, and only data that cannot identify individual participants will be shared.

## Interventions

### Explanation for the choice of comparators {6b}

The comparator in this trial is TAU only, corresponding to a waitlist control group. In both groups, participants continue their stable usual care (e.g., prescription medications or insurance-covered physical therapy) or receive no specific treatment, provided they do not meet the exclusion criteria. Treatments listed in the exclusion criteria, such as interventional procedures, medical narcotics, or intravenous infusions for pain relief, are not permitted as part of TAU. In Japan, there is no standardized definition of TAU for chronic pain in the Japanese clinical guidelines for chronic pain [14, 28]. These guidelines present graded recommendations for pharmacological, interventional, physical, and psychological treatments but do not specify a fixed package of “usual care.” Accordingly, TAU in this study is defined as the continuation of each participant’s ongoing treatment situation without initiating new interventions or changing the type, dosage, or administration of existing treatments. Participants in the intervention group receive CBT-CP in addition to TAU, while those in the control group remain on TAU only until the post-assessment at 15 ± 2 weeks after randomization. This approach is consistent with how TAU or waitlist control conditions have been treated in international trials and systematic reviews [15, 27].

### Intervention description {11a}

CBT-CP in this study is a structured and manualized eight-session program, with an original workbook and worksheet consisting of psycho-education, relaxation training, activity pacing, cognitive restructuring, and summary and relapse prevention (Table 1). Materials from the pilot version of the program have already been made freely available to healthcare professionals via the study website (in Japanese), and the present trial materials are planned to be published after study completion to support dissemination of CBT-CP in Japan. Participants will undergo eight sessions of CBT-CP, each lasting approximately 40 min, with the aim of completing the program within 14 weeks. If necessary, sessions may extend beyond the 15th week; however, the post-assessment is conducted at 15 ± 2 weeks irrespective of the intervention schedule. Homework will be provided in each session so that participants can practice the skills learned in real-life situations. The sessions are arranged face-to-face and weekly, unless inconvenient for the participant or therapist. If the therapist and participant cannot complete all the content in eight sessions and both deem it necessary, up to two additional sessions may be permitted.

**Table 1:** Outline of the CBT-CP program

Session	Components	Homework
1	Psycho-education regarding CBT and chronic pain (CBT model, self-monitoring) and goal setting	Identifying vicious cycles using the CBT model
2	Relaxation training (breathing method, progressive muscle relaxation)	Practicing relaxation
3	Activity pacing 1 (reviewing the relationship between behavior and pain)	Recording activity log
4	Activity pacing 2 (setting the pace of activity and rest periods)	Practicing the set activity
5	Activity pacing 3 (adjusting the pace, including countermeasures against obstacles to activity)	Practicing activity including obstacle countermeasures
6	Cognitive restructuring 1 (identifying and distancing cognitive factors that interfere with activity)	Identifying obstructive cognition
7	Cognitive restructuring 2 (attempting to alter cognitive factors that interfere with activity)	Distancing from and restructuring the cognition
8	Summary and relapse prevention	Continuing to use the skills that have served them well

*Abbreviations: CBT-CP* cognitive behavioral therapy for chronic pain, *CBT* cognitive behavioral therapy

In Session 1, the therapists reiterate that the purpose of the CBT-CP program is not to attain pain-free conditions but to improve QoL. Subsequently, through self-monitoring, participants practice capturing the problems caused by chronic pain using the CBT model. The CBT model shows that humans respond to their circumstances, including situations and interpersonal relationships, in four aspects—cognitive, emotional, behavioral, and physiological—which all interact with each other. Finally, the participants discuss their individual goals and hopes for improvement through CBT-CP.

In Session 2, breathing and progressive muscle relaxation techniques are implemented to relax the body and mind and experience how autonomous and intentional activities can change the four aspects of the CBT model and their interactions.

In Sessions 3–5, activity pacing, the core intervention of our CBT-CP program, will be conducted. After reviewing the relationship between behavior and pain using an activity log, participants will choose their favorite activities that they value and set the pace of the activity and rest periods. In addition, obstacles to implementing these activities will be identified and countermeasures will be taken. Activity pacing could combine other behavior change techniques, such as behavioral activation and exposure [29], and can be tailored to the participant’s chosen or preferred activity.

In Sessions 6–7, the focus will be specifically on the cognitive factors that interfere with the activities undertaken in the previous sessions. The first goal is to be aware of the factors; then, to distance oneself from them, such as through externalization; and finally, to develop skills not influenced by the factors so that the activity initiated by participants in Sessions 3–5 is maintained.

In Session 8, participants summarize their learning from CBT-CP and discuss measures to prevent recurrence.

CBT-CP is to be conducted face-to-face but is allowed to be conducted remotely only if specified conditions are met. When it is inappropriate to provide CBT-CP face-to-face to comply with regulations by the government, local governments, and implementing institutions to prevent the spread of the novel coronavirus infection, online implementation is acceptable if the requirements based on the respective revised guidelines for the appropriate implementation of telemedicine and safety management of medical information systems in 2018 and 2017, by the Japanese Ministry of Health, Labour and Welfare, are met. This contingency applies only to such official regulations and not to individual-level infection risk. Online implementation is limited to the period during which the regulation is imposed, and face-to-face implementation will be resumed as soon as the regulation is lifted. In addition, it is permitted only if predefined requirements are met, such as approval from the attending physician, no recent history of requiring emergency face-to-face care, access to appropriate equipment and secure internet at the participant’s own expense, availability of a quiet private space, and the establishment of a safety plan in advance (e.g., emergency contacts and procedures).

### Criteria for discontinuing or modifying allocated interventions {11b}

Participants who meet any of the following discontinuation criteria will be excluded from the study:

- Declines to participate in the study or withdraws consent
- No contact for more than 1 month. This is defined as failure to attend scheduled sessions and lack of response to repeated contact attempts (e.g., phone calls or study-related emails) from therapists or medical staff during that period.
- Difficulty in continuing the study owing to a serious adverse event
- Did not meet the eligibility criteria at the enrollment stage
- The entire study is terminated
- The principal investigator and research collaborators determine that it is appropriate to terminate participation in the study for other reasons

Notably, CBT-CP is designed to be completed within 14 weeks, but sessions may occasionally extend beyond the 15th week. Such extensions are not considered protocol violations, provided that the post-assessment is conducted at 15 ± 2 weeks.

If the Ethical Review Board recommends or directs discontinuation of the study, it will be terminated. Additionally, the principal investigator will consider whether to continue the study when any of the following events occur:

- Critical information is obtained regarding the quality, safety, or efficacy of the treatment
- The Ethical Review Board instructs the investigator to change the protocol and it is deemed difficult to accept the change

### Strategies to improve adherence to interventions {11c}

To promote adherence, participants’ willingness and confidence to complete homework assignments are rated on an 11-point scale at each session, and the significance of the homework is reinforced or its content is adjusted as appropriate. With participants’ consent, session audio recordings and copies of completed worksheets are used in supervision to ensure treatment fidelity. Therapists complete a standardized session checklist to record how many of the planned items were implemented in each session and receive supervision at least twice per case, with additional consultation as needed to ensure safety. Supervision is provided by HH, TF, KI, and Y. Takagishi, the program developers with over 8 years of experience in CBT supervision, and consultation is provided by MH, the overall supervisor of this program, who has more than 20 years of experience in CBT supervision and consultation.

### Relevant concomitant care permitted or prohibited during the trial {11d}

To appropriately evaluate the efficacy of the intervention, concomitant care is restricted during the study period. Participants are permitted to continue their stable usual care, such as prescription medications or physical therapy, provided that the type, dosage, and administration remain unchanged. In contrast, they are asked not to (1) initiate other psychotherapy or participate in other clinical trials; or (2) begin medical examinations or treatments that meet the exclusion criteria, such as interventional pain procedures, medical narcotics, or intravenous infusions for pain relief. If a change in usual care is medically necessary, this takes priority, and the participant is instructed to report the change at the next visit. When participants have an attending physician at another hospital, a written summary of the study requirements is also sent to the physician to request that the participant’s usual care remain unchanged from enrollment until the post-assessment.

### Provisions for post-trial care {30}

CBT-CP in this study is limited to eight sessions and will not continue after trial completion. Thereafter, participants will continue to receive usual care from their attending physicians. In the unlikely event of harm related to trial participation, appropriate medical treatment will be provided within the framework of routine clinical care under the Japanese health insurance system. As this trial involves only minimal risk—CBT-CP is delivered as an adjunct to usual care and is not expected to cause serious adverse events—no financial compensation or additional allowances will be provided. Participants are informed of these provisions in advance and give their consent accordingly.

### Outcomes {12}

#### Primary outcome

The primary outcome is health-related QoL, assessed using the EQ-5D-5L [30]. The Japanese version, which has a reliable and valid Japanese scoring system [31], is used. Scores range from 0 (death) to 1 (full health).

#### Secondary outcomes and psychosocial variables

Secondary outcomes include a range of psychosocial variables, which may serve not only as outcomes responsive to CBT-CP but also as potential mediators or moderators of change in QoL, the primary outcome. These are assessed as follows:

- Health-related QoL (secondary measure): Mental and physical health-related QoL is assessed using the Medical Outcomes Study 12-Item Short Form Health Survey (SF-12), which yields physical and mental component summary scores [32]. In Japan, the inclusion of a role/social component summary has also been recommended and validated [33]. These components are calculated based on the 2007 national norms, with scores ranging from 0 to 100 [34]. Higher scores indicate better health status.
- Pain severity: Pain severity is assessed using the numerical rating scale (NRS), with 0 indicating no pain and 10 indicating the worst imaginable pain [35]. Participants rate their pain experienced in the last week as follows: (1) maximum, (2) minimum, (3) average, and (4) current. For analysis, the average score is used to maximize reliability [35, 36]. Higher scores indicate greater pain severity.
- Disability: Disability due to chronic pain is assessed using the Pain Disability Assessment Scale (PDAS) [37, 38], which evaluates limitations in physical exercise and movement. Its reliability and validity have been reported [37, 38]. Higher scores indicate greater disability.
- Depressive symptoms: The Patient Health Questionnaire-9 (PHQ-9) is used to assess depressive symptoms common in primary care, according to the Diagnostic and Statistical Manual of Mental Disorders [39]. The Japanese version is reliable and valid [40]. Higher scores indicate poorer mental health.
- Pain catastrophizing: The Pain Catastrophizing Scale (PCS) is used to assess cognitive factors that sustain chronic pain [41, 42]. The Japanese version is reliable and valid [43], with higher scores indicating greater catastrophizing.
- Fear of movement: Fear of movement is assessed using the Japanese version of the Tampa Scale for Kinesiophobia 11 (TSK-11), which has sufficient reliability and validity [44]. Higher scores indicate greater fear of movement.
- Pain self-efficacy: Pain-related self-efficacy is assessed with the Pain Self-Efficacy Questionnaire (PSEQ) [45]. The Japanese version is reliable and valid [46]. Higher scores indicate greater confidence in performing activities despite pain.

In addition, two further variables are included as potential moderators:

- Prognostic risk: The Keele STarT Back Screening Tool generic condition (STarT Back) [47], which assesses physical and psychosocial prognostic factors for chronic pain. The Japanese version is reliable and valid [48, 49]. Scores classify patients into three prognostic risk levels.
- Somatic symptom burden: The Somatic Symptom Scale-8 (SSS-8) [50], which assesses the overall burden of somatic symptoms. The Japanese version is reliable and valid [51]. Higher scores indicate greater burden of physical symptoms.

#### Demographic and clinical characteristics

Demographic and clinical characteristics are collected at the initial evaluation using a standardized background checklist. Eligibility criteria are first confirmed and recorded. Data collected include:

- Demographic information, such as age, sex, education, family structure, and employment status.
- Clinical information, such as pain site, diagnosis, pain duration, medical history, and current clinical condition.
- Treatment history and status, such as previous and current pain treatments, medication use, and related healthcare utilization.

#### Process measures

At the start of each visit, physicians, therapists, or a research assistant verbally check for adverse events and any changes in usual care. During the intervention period, at the beginning of each session, participants rate their sense of completeness, average pain intensity, and sense of coping with pain in the last week on an 11-point scale (0–10) by the therapist.

### Participant timeline {13}

**Fig. 1:**
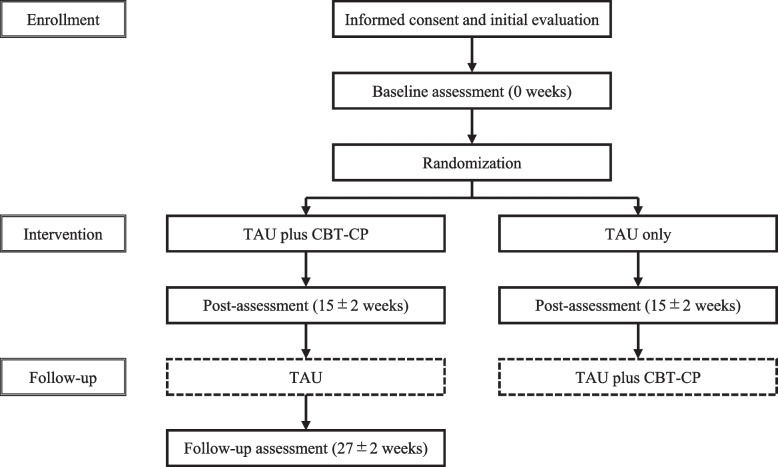
CONSORT diagram for this clinical trial. *Abbreviations: CBT-CP* cognitive behavioral therapy for chronic pain, *TAU* treatment as usual. Post- and follow-up assessments are conducted in person at the participating hospital. If participants cannot attend within the assessment window, they may complete a mailed questionnaire or respond by telephone

The participant timeline is shown in Fig. 1. After providing informed consent, participants undergo an initial evaluation, followed by a baseline assessment within 4 weeks (defined as 0 weeks for the trial schedule). Eligible participants are then enrolled and randomized in a 1:1 ratio to the intervention group (TAU plus CBT-CP) or the waitlist control group (TAU only).

Randomization is performed centrally using an electronic data capture (EDC) system (HOPE eACReSS; Fujitsu Ltd.), managed by the Translational Medical Center of the National Center of Neurology and Psychiatry. Stratified block randomization is applied by hospital (study site). To emphasize transparency, registration and randomization are usually performed in front of eligible participants on a study computer, unless there is a specific reason not to do so.

Both groups undergo a post-assessment at 15 ± 2 weeks. The intervention group receives CBT-CP with the goal of completing all eight sessions within 14 weeks. If the intervention extends beyond the 15th week, the post-assessment is nevertheless conducted at 15 ± 2 weeks as scheduled. A follow-up assessment at 27 ± 2 weeks is conducted only for the intervention group to explore mid-term effects because the timing of CBT-CP initiation for the waitlist control group depends on post-assessment findings and/or social circumstances, making follow-up assessment impractical. Although collecting follow-up data for the waitlist control group would have been desirable, it was judged unfeasible to obtain consistent data under these circumstances.

All assessments are in principle conducted in person at the participating hospital. However, for the post-assessment and follow-up assessment, if participants are unable to attend within the assessment window (± 2 weeks), they may instead complete a mailed questionnaire or provide responses by telephone.

### Sample size {14}

In the preceding pilot study, among participants with baseline EQ-5D-5L ≤ 0.80 (12 of 15), Hedges’ g for EQ-5D-5L from baseline to post-treatment was − 1.24 (90% confidence interval [CI]: − 2.14 to − 0.34), with the negative sign reflecting model coding (improvement); the corresponding mean difference was 0.18 (90% CI: 0.09 to 0.27) [22]. Because this was a within-participants effect size without a control group, it is treated only as a reference value. For between-participant comparisons of QoL, a meta-analysis reported a CI ranging from − 0.74 to 0.11 for the effect size of CBT-CP [52]. In addition, an RCT in patients with low pain intensity yielded an effect size of 0.23 [53], whereas an RCT in patients with high pain intensity reported an effect size of 1.92 [54], suggesting a dose-response relationship between baseline pain intensity and treatment effect [55].

Based on these findings, we set the anticipated effect size for this trial at 0.74, the upper limit reported in the meta-analysis [52], because this study targets patients with relatively high chronic pain intensity. The dropout rate was assumed to be 20%, and the correlation between time points for the primary outcome was set at 0.50, based on the pilot study [22]. With a two-sided significance level of 0.05 and a statistical power of 0.90, the required sample size for a linear mixed model (LMM) analysis [56] was calculated as 30 participants per group. Accordingly, the target total sample size is 60 participants, with 30 in each arm.

### Recruitment {15}

Participants are primarily recruited from the outpatient clinics of the participating hospitals, where physicians approach potentially eligible patients during routine clinical visits. Those who express interest are then provided with written information about the study. In addition to this direct recruitment, participants may also be referred by colleagues or affiliated institutions, or may contact the hospital themselves after finding information about the study on the study website. To enhance the likelihood of reaching the target sample size, the trial is organized as a multicenter collaboration across seven hospitals in Japan, thereby enabling recruitment from a broader patient base.

At the same time, opportunities to provide CBT-CP in routine practice are still limited in Japan. Clinical care understandably takes priority in everyday hospital settings, and in many departments that treat chronic pain, psychologists are not employed at all, even on a part-time basis. Even when a psychologist is present, they are not necessarily able to deliver CBT-CP, either because their clinical hours are very limited or because their therapeutic orientation is not CBT. In this trial as well, the same constraints apply: CBT-CP is provided mainly by therapists qualified to deliver it—most of whom are university faculty collaborating on the study—and they travel to the participating hospitals to conduct the intervention only when eligible participants have been recruited. Because their primary academic duties remain at their home institutions, their availability is restricted to a small portion of time, typically ranging from a half day to 1 day per week. For these reasons, large-scale advertising or mass recruitment is not feasible, and enrolment is being pursued gradually through clinical encounters and professional referrals.

## Assignment of interventions: allocation

### Sequence generation {16a}

Participants are allocated in a 1:1 ratio to the intervention or waitlist control group. Randomization is performed centrally using an EDC system. To avoid imbalance in group allocation across hospitals, stratified block randomization is used, with the seven participating hospitals (study sites) as stratification factors. Details of block size are concealed to maintain allocation unpredictability.

### Concealment mechanism {16b}

The allocation sequence is embedded within the central EDC system and is inaccessible to all study personnel and participants. Registration and randomization are conducted electronically, ensuring that allocation is not revealed until the point of assignment, thereby maintaining strict concealment of the sequence.

### Implementation {16c}

The randomization sequence is generated automatically by the EDC system. At each hospital, collaborating physicians are responsible for recruiting and registering eligible participants, with assistance from therapists or a research assistant when needed, always under the physician’s supervision. Once registration is completed, the EDC system assigns participants to groups. To emphasize transparency, randomization is usually performed in front of participants using the study computer, unless specific circumstances make this impractical.

## Assignment of interventions: blinding

### Who will be blinded {17a}

Because the intervention involves adding CBT-CP to TAU, masking participants, physicians, therapists, and a research assistant is not feasible. Outcomes are assessed exclusively using self-administered questionnaires; therefore, independent outcome assessors are not required. Data analysts work with de-identified datasets with coded group labels. Because the intervention and waitlist control groups are inherently distinguishable, blinding cannot be maintained; however, the standardized manualized program, structured therapist training, supervision, and fidelity procedures help ensure consistent delivery across groups.

### Procedure for unblinding if needed {17b}

Blinding is not applied in this trial; thus, no unblinding procedures are required. Because the intervention and waitlist control groups are inherently distinguishable, accidental unblinding is not an issue.

## Data collection and management

### Plans for assessment and collection of outcomes {18a}

As shown in Table 2, the primary outcome, secondary outcomes and psychosocial variables, demographic and clinical characteristics, and process measures are assessed. Outcomes and psychosocial variables are self-reported using A4 booklet-format questionnaires. Demographic and clinical characteristics at the initial evaluation, as well as process measures during CBT-CP sessions, are obtained verbally by collaborating physicians, therapists, or a research assistant and recorded on standardized forms.

**Table 2:** Schematic diagram for the assessment schedule (SPIRIT Fig. 2013)

Time points	Initial evaluation	Baseline assessment	Intervention period	Post-assessment	Follow-up assessment
Visit	1	2	3–10	11	12
Week	−4–0	0	1–14	15 ± 2	27 ± 2
Demographic and clinical characteristics					
Eligibility criteria	V				
Demographic information	V				
Clinical information	V				
Treatment history and status	V				
Primary outcome					
EQ-5D-5L		S		S	S
Secondary outcomes and psychosocial variables					
SF-12		S		S	S
NRS		S		S	S
PDAS		S		S	S
PHQ-9		S		S	S
PCS		S		S	S
TSK-11		S		S	S
PSEQ		S		S	S
STarT Back		S			
SSS-8		S			
Process measures					
Adverse events		V	V	V	V
Changes in usual care		V	V	V	V
Sense of completeness			V		
Average pain intensity			V		
Sense of coping with pain			V		

*Abbreviations and codes*: *S* self-reported by participant, *V* verbally obtained by a physician, therapist, or research assistant, *EQ-5D-5L* EuroQol five-dimensional questionnaire five-level, *SF-12* Medical Outcomes Study 12-Item Short Form Health Survey, *NRS* numerical rating scale, *PDAS* Pain Disability Assessment Scale, *PHQ-9* Patient Health Questionnaire-9, *PCS* Pain Catastrophizing Scale, *TSK-11* Tampa Scale for Kinesiophobia 11, *PSEQ* Pain Self-Efficacy Questionnaire, *STarT Back* Keele STarT Back Screening Tool generic condition, *SSS-8* Somatic Symptom Scale-8

To ensure data quality, all questionnaires are compiled into a booklet that participants complete independently. After completion, collaborating physicians or therapists check for missing responses. Data are then entered item by item into the predefined format of the EDC system, which ensures that any missing responses are identified again during entry. In addition, the system includes range checks to prevent invalid values and automatically calculates scale scores, further reducing the risk of human error. Data entry personnel are also instructed to recheck entries after a time interval to minimize input errors. For verbally collected data, the use of standardized forms and subsequent entry into the EDC system similarly ensures that any omissions are identified at the time of data entry. Because these procedures are straightforward and standardized, no additional training workshop is considered necessary.

Because participants in both the intervention group and the waitlist control group are recruited within the same hospitals, performance bias due to differential expectations of treating physicians cannot be entirely excluded. However, therapists only meet participants when delivering CBT-CP, minimizing the risk of therapist-related bias, and participants in the waitlist control group receive CBT-CP only after the post-assessment, preventing differential treatment during the assessment period. In addition, attending physicians at other hospitals are asked to keep TAU unchanged until the post-assessment. Further measures to reduce bias include the use of a manualized intervention program, structured therapist training and supervision, and treatment fidelity procedures, which are detailed in Items 10, 11a, 11c, and 11d.

### Plans to promote participant retention and complete follow-up {18b}

To promote retention, session schedules (timing and rescheduling) are arranged flexibly, and CBT-CP is provided face-to-face under ordinary circumstances. However, when sessions cannot be held in person owing to regulations by the government, local governments, or implementing institutions, online implementation is also permitted under predefined safety requirements (see Item 11a). Similarly, for the post-assessment and follow-up assessment, if participants are unable to attend within the assessment window, they may complete a mailed questionnaire or provide responses by telephone (see Item 13). Therapists also encourage engagement by reviewing and adjusting homework as appropriate, thereby supporting adherence throughout the intervention.

For participants who discontinue or withdraw from the study, outcome data are still collected at post-assessment, and at follow-up for the intervention group, whenever possible. All available data are included in the analysis according to the intent-to-treat (ITT) principle.

### Data management {19}

Participants’ data are anonymized at each hospital and entered into the EDC system using research IDs, with personal identifiers removed. Paper-based materials such as consent forms, demographic checklists, and completed questionnaires, as well as electronic audio files for supervision, are securely stored in locked cabinets or password-protected drives within each hospital. All such materials are destroyed in an unidentifiable form 5 years after study completion or 3 years after publication, whichever is later. Data management is conducted with careful attention to transparency and future reusability, with anonymized datasets made available for secondary analyses upon appropriate request and following approval by the relevant ethics committee.

### Confidentiality {27}

Research data are coded with research IDs, and linkage to personal identifiers is kept only within each hospital under restricted access. Identifiable information is never shared outside the hospitals. When electronic audio files are temporarily transferred for supervision, a secure, access-restricted online platform is used, and files are promptly deleted after transfer.

### Plans for collection, laboratory evaluation, and storage of biological specimens for genetic or molecular analysis in this trial/future use {33}

No biological specimens are collected in this trial.

## Statistical methods

### Statistical methods for primary and secondary outcomes {20a}

A detailed statistical analysis plan (SAP) will be developed and finalized prior to database lock.

The main analysis will estimate the effect size for the primary outcome (EQ-5D-5L) using LMM [64] based on the ITT principle. The LMM method is selected because of its strength in handling missing data and its ability to incorporate random effects. The analyses will be conducted with the outcome scores at each assessment time point as the dependent variable, time (baseline and post-assessment), group (intervention vs. waitlist control), and interaction between time and group as fixed effects, and participants as random effects. No special treatment will be applied to discontinued cases or missing values for ITT analysis, but as a sensitivity analysis, best- and worst-case scenarios will be examined by imputing hypothetical maximum and minimum plausible values based on the ranges of each scale, defined a priori and prespecified in the SAP. To address the potential non-normality and ceiling effects of the EQ-5D-5L, alternative modeling approaches (e.g., generalized linear models, beta regression) will also be considered in the SAP. Two-sided *p* values < 0.05 will be considered statistically significant.

Secondary analyses will estimate effect sizes for multiple psychosocial outcomes using the same LMM framework. In addition, the intervention group will undergo a follow-up assessment at 27 ± 2 weeks after randomization, and the mid-term effect of CBT-CP will be explored in a single-arm, pre/post comparison. Because multiple secondary outcomes (approximately 11 variables) will be analyzed, type I error will be controlled using Bonferroni correction. However, given the limited sample size, this conservative adjustment is expected to markedly reduce statistical power. Therefore, unadjusted results will be reported as the primary descriptive findings, while results with Bonferroni correction will also be presented to indicate the potential risk of type I error. Effect sizes and CI will be reported to aid interpretation, and all analyses of secondary outcomes will be considered exploratory and primarily hypothesis-generating.

Exploratory analyses will examine whether psychosocial variables, participant characteristics, and process measures function as potential moderators or mediators of treatment effects.

### Interim analyses {21b}

No interim analysis will be performed, and no formal stopping rules are planned. This decision reflects the low anticipated risk of CBT-CP and the modest sample size. Any deviations from this policy, if needed, will be prespecified in the SAP.

### Methods for additional analyses (e.g., subgroup analyses) {20b}

Preplanned subgroup analyses are not powered and will therefore be exploratory only. If conducted, they will examine effect modification by prespecified baseline factors such as age group and pain site. Interaction terms may be added to the LMM, and sensitivity analyses for subgroup or secondary outcome models may also be considered. These additional analyses will be exploratory and interpreted cautiously, with all statistical details, including handling of multiple comparisons, prespecified in the SAP.

### Methods in analysis to handle protocol non-adherence and any statistical methods to handle missing data {20c}

The primary analysis will follow the ITT principle. Protocol non-adherence will not lead to exclusion from the ITT set; per-protocol analyses are not planned. Missing outcome data will be handled within the LMM framework, and no special imputation is applied for the primary analysis. As sensitivity analyses, best-case and worst-case scenarios will be examined using hypothetical values defined a priori and prespecified in the SAP.

### Plans to give access to the full protocol, participant-level data, and statistical code {31c}

The protocol is publicly available through the University Hospital Medical Information Network Clinical Trials Registry (UMIN000042798). This article provides the full protocol with additional methodological details beyond what is available in the registry. In line with open science practices, the finalized SAP will also be made publicly available through the same registry before database lock.

After publication of the main results, de-identified participant-level data, the associated data dictionary, and statistical code will be made available upon reasonable request to the corresponding author, following approval by the relevant ethics committee. Intervention materials, including the CBT-CP workbook and therapist manual, are planned to be published after study completion to support dissemination in Japan.

## Oversight and monitoring

### Composition of the coordinating center and trial steering committee {5d}

The coordinating center for this trial is located at Kansai University, led by the principal investigator, who is responsible for overall trial management and day-to-day coordination. Although no independent trial office is established, the principal investigator directly oversees all organizational aspects of the study. In addition, a research assistant at the National Center of Neurology and Psychiatry assists with tasks related to the EDC system and data coordination.

Oversight of the trial is provided through a Trial Steering Committee convened every 2 months. Attendance is prioritized to ensure that the principal investigator and the site principal investigators (or, when necessary, a delegate from each site such as a therapist) are present. However, all trial team members are invited to participate, and attendance by additional investigators or therapists beyond the site principal investigators is welcomed. The committee reviews recruitment progress, enrollment numbers, reasons for discontinuation or dropout, adverse events, and data entry status, and also serves as the forum for discussing and, when necessary, adjudicating issues such as the interpretation of inclusion/exclusion criteria, given the heterogeneous clinical profiles of patients with chronic pain.

Day-to-day project management is conducted by the principal investigator at Kansai University, with technical support for the EDC system provided by the Translational Medical Center of the National Center of Neurology and Psychiatry. At each study site, the site principal investigator (a collaborating physician) holds ultimate responsibility for local trial conduct, including patient recruitment, informed consent, and adherence to protocol procedures.

No separate stakeholder or public involvement group has been established for this trial.

### Composition of the data monitoring committee, its role and reporting structure {21a}

No independent data monitoring committee has been established for this trial because the intervention (CBT-CP) is a non-invasive psychological therapy. Although some questionnaires include sensitive items such as suicidal ideation and self-injurious behavior, the trial is classified as involving only minor invasions, and according to ethical and regulatory standards in Japan, a formal committee of this kind is not required.

Instead, oversight of safety and trial conduct is ensured through the structures described in Item 5d. In brief, a Trial Steering Committee meets every 2 months to review recruitment, dropouts, adverse events, and data entry. Serious adverse events are immediately shared with all trial team members via a mailing list, and appropriate action is coordinated. In addition, the principal investigator and a data coordinator monitor the electronic data capture system for missing data or deviations, while each site principal investigator checks document retention, data accuracy, and consent procedures, initiating additional monitoring as needed in accordance with institutional regulations.

### Adverse event reporting and harms {22}

Although CBT-CP is classified as involving only minor invasions and no serious adverse events attributable to the intervention are anticipated, adverse events will be systematically monitored at every visit. At each visit, physicians, therapists, or a research assistant will verbally check for any adverse events, and participants may also report events spontaneously at any time.

All adverse events and serious adverse events will be recorded and reviewed from a clinical perspective, including considerations of their seriousness and potential relationship to the intervention. These judgments will be made jointly by the principal investigator and the site principal investigator, with precedence given to the clinical judgment of the site. Any serious adverse event will be promptly reported to the head of the institution and to the institutional ethics committee, as required by institutional and national regulations, and will also be shared with all trial team members to coordinate an appropriate response. An ad hoc meeting may be convened if necessary to determine implications for the trial.

At the site level, each site principal investigator is responsible for ensuring that source documents are retained and that reporting procedures are properly implemented. Clinical significance will always be prioritized in deciding whether an individual participant continues treatment, with decisions made in consultation with the site principal investigator and the attending physician.

### Frequency and plans for auditing trial conduct {23}

Because this study involves only a psychological intervention classified as involving minor invasions, and because in Japan such trials are in principle not required to undergo monitoring or auditing, no independent audit of trial conduct is planned. Instead, oversight is maintained through meetings of all trial team members held every 2 months, during which trial progress and safety issues (e.g., recruitment, discontinuations, and adverse events) are reviewed, and through ongoing monitoring of data entry within the EDC system. Each site principal investigator also checks source documents and consent procedures in accordance with institutional regulations, and institutional ethics committees review trial conduct as required.

### Plans for communicating important protocol amendments to relevant parties (e.g., trial participants, ethical committees) {25}

Any important protocol amendments will be reviewed and approved by the institutional ethics committees before implementation, updated in the trial registry, and communicated to all trial team members. When amendments may affect participants’ willingness to continue, updated information will be provided and re-consent will be obtained as required.

### Dissemination plans {31a}

The results of this trial will be presented at relevant academic conferences and published in peer-reviewed journals. To facilitate dissemination to healthcare professionals, the CBT-CP workbook and therapist manual will be published after study completion. In addition, information on publications, conference presentations, workshops, and related activities by the research team is regularly updated on the publicly accessible study website (every 2 months), which can be viewed by researchers, healthcare professionals, and patients. Although no separate lay summary or dedicated patient workshops are currently planned, the dissemination of trial materials and open access to the study website are intended to ensure that patients and the wider public can access information about the trial and its outcomes.

## Discussion

### Overview and contributions

This multicenter randomized controlled trial evaluates the face-to-face, eight-session, individualized CBT-CP program added to TAU in Japan, with QoL as the primary outcome, compared with TAU only. Distinctive features include (1) prioritizing QoL as the primary outcome; (2) a brief, manualized intervention delivered with specified therapist training, supervision, and fidelity procedures; and (3) a multicenter design across multiple Japanese sites to enhance generalizability within Japan. A prior Japanese RCT implemented a single-center, videoconference-based integrated CBT program over 16 sessions, with pain intensity as the primary outcome, and it included a cost-utility analysis [26]. In contrast, the present trial focuses on face-to-face delivery in routine clinical settings, narrows the intervention elements to a practical core (psycho-education, relaxation, activity pacing, and cognitive restructuring), and halves the number of sessions. By limiting components and session count, therapist training and program implementation become more feasible within current Japanese service capacity, potentially accelerating dissemination.

### Comparator and Japanese practice

We adopted a CBT-CP added to TAU versus TAU only design. In Japan, TAU for chronic pain is heterogeneous and not standardized; estimating the incremental efficacy of adding CBT-CP to stable usual care aligns with our pragmatic aim to quantify its added benefit and is consistent with routine clinical practice in Japan. Although CBT-only or other designs can be valuable for mechanism-focused efficacy questions, the present trial addresses a policy-relevant question for Japanese care pathways: does adding CBT-CP to what patients already receive improve QoL? Future work in Japan could complement these findings by using active psychological control conditions to isolate specific treatment effects where appropriate.

### Secondary and exploratory analyses

Beyond QoL, secondary outcomes will characterize broader changes in functioning and mental health, drawing on a predefined set of seven validated scales, with some analyses using lower-order subscales where appropriate, to capture domains such as pain intensity, pain-related disability, and psychological factors related to chronic pain. Given multiplicity and a modest sample, these analyses will be interpreted as exploratory, with effect sizes and confidence intervals reported, and multiplicity-aware analyses conducted as prespecified in the SAP. Exploratory moderator and mediator analyses will use the same pool of psychosocial measures together with two prespecified moderators that are not modeled as outcomes, such as prognostic risk stratification and overall somatic symptom burden, to generate hypotheses about for whom and through which processes CBT-CP may confer benefit in Japan.

### Strengths, limitations, and implications

Strengths include the multicenter design within Japan; a brief, manualized CBT-CP compatible with current service capacity; prioritization of patient-reported QoL; specified therapist eligibility with supervised certification prior to implementation and ongoing fidelity checks; centralized concealed randomization; and analytic transparency through a prespecified SAP, with robustness assessments considered where appropriate.

Several limitations warrant consideration. Blinding of participants and therapists is infeasible in trials of psychological interventions; reliance on self-report outcomes may introduce expectation effects, although the waitlist control group receives CBT-CP only after post-assessment, and analysts work with anonymized datasets. The sample size, together with multiple outcomes, limits power for secondary outcomes and moderator and mediator models; these findings will be hypothesis-generating. Exclusion criteria, such as postsurgical or injury-related pain, active psychosis or mania, and concurrent psychotherapy or interventional pain procedures, constrain generalizability. Mixed pain conditions are included while primary headache is excluded; heterogeneity may attenuate condition-specific effects, yet is consistent with routine clinical practice in Japan, where clinics manage diverse presentations. Finally, only the intervention arm undergoes a 27 ± 2-week follow-up to explore mid-term durability; this prevents between-group comparisons at follow-up and will be clearly labeled as single-arm pre/post evidence.

Although the intervention is intended to be completed within 14 weeks, sessions may extend beyond the 15th week in practice; however, the post-assessment is strictly scheduled at 15 ± 2 weeks, ensuring consistent outcome measurement regardless of intervention timing.

## Conclusion

This trial estimates the incremental efficacy of adding a brief, fidelity-assured, face-to-face CBT-CP program to TAU in Japan, with QoL as the primary outcome within routine Japanese care. Findings are expected to guide clinical adoption, workforce development, and implementation models within Japan, with potential relevance to other Asian settings as subsequent work accumulates.

### Trial status

This manuscript is based on protocol version 7 (approved on July 13, 2023), which was the version active at the time of initial submission. Recruitment began on February 12, 2021, and was originally planned to be completed by March 31, 2026. At submission, 30 participants (50%) were enrolled.

Since then, the protocol has been amended to version 10 (approved on April 16, 2025), with the recruitment period extended to March 31, 2027. As of September 2025, 40 participants (67%) have been enrolled.

## Data Availability

A summary protocol is publicly available in the University Hospital Medical Information Network Clinical Trials Registry (UMIN000042798), and this article provides the full protocol with additional methodological details. In addition, the finalized SAP will be made available through the same registry before database lock. After completion of the study and publication of the main trial results, the final trial dataset may be available from the principal investigator (HH) upon reasonable request, subject to approval by the relevant ethics committee in accordance with the latest ethical guidelines. No contractual agreements exist that limit access for investigators.

## Abbreviations

ADL: Activities of daily living
CBT: Cognitive behavioral therapy
CBT-CP: Cognitive behavioral therapy for chronic pain
CI: Confidence interval
EDC: Electronic data capture
EQ-5D-5L: EuroQol five-dimensional questionnaire five-level
ITT: Intent-to-treat
LMM: Linear mixed model
NRS: Numerical rating scale
PCS: Pain Catastrophizing Scale
PDAS: Pain Disability Assessment Scale
PHQ-9: Patient Health Questionnaire-9
PSEQ: Pain Self-Efficacy Questionnaire
QoL: Quality of life
RCT: Randomized controlled trial
SAP: Statistical analysis plan
SF-12: Medical Outcomes Study 12-Item Short Form Health Survey
SMD: Standardized mean difference
SSS-8: Somatic Symptom Scale-8
STarT Back: Keele STarT Back Screening Tool generic condition
TAU: Treatment as usual
TSK-11: Tampa Scale for Kinesiophobia 11
